# Digital Health Literacy of Children and Adolescents and Its Association With Sociodemographic Factors: Representative Study Findings From Germany

**DOI:** 10.2196/69170

**Published:** 2025-05-05

**Authors:** Lisa Stauch, Denise Renninger, Pia Rangnow, Anja Hartmann, Lisa Fischer, Kevin Dadaczynski, Orkan Okan

**Affiliations:** 1 Department of Sport and Health Sciences TUM School of Medicine and Health Technical University of Munich Munich Germany; 2 WHO Collaborating Centre for Health Literacy TUM School of Medicine and Health Technical University of Munich Munich Germany; 3 Department of Applied Health Sciences Bochum University of Applied Sciences Bochum Germany; 4 Department of Health Sciences Fulda University of Applied Sciences Fulda Germany; 5 Department of Medicine Institute of Health Sciences University of Lübeck Lübeck Germany; 6 Department of Sports and Health Sciences Faculty of Human Science University of Potsdam Potsdam Germany; 7 Centre for Applied Health Science Leuphana University of Lüneburg Lüneburg Germany

**Keywords:** digital health literacy, sociodemographic, health promotion, child and adolescent health, cross-sectional

## Abstract

**Background:**

Children and adolescents extensively use the internet in their daily lives, often seeking information related to health and well-being. In modern society, the volume of health information available in digital environments is constantly increasing. This includes both reliable and misleading content, making it challenging to assess trustworthiness. Digital health literacy is essential for navigating the digital information ecosystem, protecting oneself from misinformation, and making informed health decisions.

**Objective:**

This representative study aims to examine the digital health literacy of children and adolescents in Germany and its association with sociodemographic factors.

**Methods:**

A cross-sectional study design with face-to-face interviews was utilized to collect data from 1448 children and adolescents aged 9-18 years in Germany between October and November 2022. Digital health literacy was assessed using an adapted and translated version of the Digital Health Literacy Instrument (DHLI), which comprises 7 subscales: operational skills, navigation skills, information searching, self-generated content, evaluating reliability, protecting privacy, and determining relevance. Bivariate and binary logistic regression analyses were conducted to examine associations between digital health literacy subscales and sociodemographic characteristics (sex, age, migration background, school type, and perceived family affluence).

**Results:**

The study found that 419 out of 1362 (30.76%) children and adolescents had a problematic level of digital health literacy, while 63 out of 1362 (4.63%) had an inadequate level. Overall, the least difficulties were observed in operational skills and determining relevance, whereas the greatest challenges were related to protecting privacy and navigation skills. Age was significantly associated with 6 of the 7 subscales (excluding protecting privacy), with younger children (9-11 years) facing a higher risk of limited skills (operational skills: odds ratio [OR] 5.42, *P*=.002; navigation skills: OR 4.76, *P*<.001; information searching: OR 4.68, *P*<.001; adding self-generated content: OR 7.03, *P*<.001; evaluating reliability: OR 3.82, *P*<.001; and determining relevance: OR 4.76.42, *P*<.001). Migration background was associated with fewer limited digital health literacy skills, while low perceived family affluence was associated with more limited skills. In the subscales of information searching, self-generated content, and evaluating information reliability, a lower risk of limited skills was observed among those with a 2-sided migration background (information searching: OR 0.62, *P*=.02; adding self-generated content: OR 0.30, *P*=.003; and evaluating reliability: OR 0.66, *P*=.03). By contrast, a higher risk was found among those with low perceived family affluence, including in the subscale of determining relevance (information searching: OR 2.18, *P*<.001; adding self-generated content: OR 1.77, *P*=.01; evaluating reliability: OR 1.67, *P*<.001; and determining relevance: OR 1.58, *P*<.001). Although school type was not associated with any dimension, sex was linked to operational skills, with females having an increased risk of limited skills (OR 1.58, *P*=.03).

**Conclusions:**

The results highlight a strong need for interventions to improve digital health literacy among children and adolescents, particularly in protecting privacy, navigation skills, and evaluating the reliability of health information. Effective interventions should be tailored to address the varying needs associated with age, migration background, and family affluence.

## Introduction

### Background

Digital media, particularly social media, are extensively utilized by children and young people, forming essential components of their everyday lives [1]. More than 80% of adolescents aged 11-18 years spend 1-4 hours per day on the internet [2]. They use digital media not only for entertainment purposes but also to obtain information about health [1,3], including topics such as mental, physical, and sexual health; nutrition and food; and information on infectious and chronic diseases [3,4]. Although they spend a large proportion of their free time online, their ability to navigate and evaluate health information, which corresponds to digital health literacy [5], remains largely unclear due to a lack of evidence [6]. Concurrent with broader technological advancements, digital transformation has increased the role of digital and commercial determinants in the health promotion, disease prevention, and health care of children and adolescents [7,8].

In modern society, individuals are exposed to disinformation and misinformation within their complex information ecosystems and environments, potentially compromising their ability to distinguish factual content from fabricated or distorted narratives [9]. The information ecosystem is highly complex due to its inherently dynamic nature and includes a diverse range of digital environments, various forms of digital media, and physical and social environments through which individuals acquire and process information [9]. The increasing proliferation of false and misleading information [10] puts children and adolescents at risk, significantly jeopardizing their health by fostering unhealthy eating habits and other detrimental health behaviors [1,11]. A recent representative study found that three-quarters of young people aged 14-24 years in Germany were exposed to misinformation weekly during the COVID-19 pandemic, with some encountering it several times a day [12]. The growing “information pollution” or “infodemic” [13] poses significant challenges to cognitive processing, decision-making, and maintaining health and well-being, while digital health literacy is considered an asset in counteracting misinformation and safely using internet-based health information [9].

The integration of digital literacy and health literacy has led to the concept of digital health literacy, defined as the capacity to access, evaluate, and utilize digital health information, as well as the proficient and appropriate use of digital technologies [14]. However, contemporary literature presents multiple definitions and understandings of digital health literacy, sometimes referred to as eHealth literacy [15]. The definition of eHealth literacy based on Norman and Skinner’s Lily model [16] is widely used in empirical research [17-19]. This model comprises 6 literacy dimensions, which can be categorized into analytic and context-specific skills [16]. This model has been criticized for its individualistic orientation and its neglect of contextual and communicative dimensions [14]. In response to the ubiquity of digital technologies in contemporary society (eg, the internet, smartphones, and apps), van der Vaart and Drossaert [5] expanded the conceptualization of eHealth literacy by developing a more comprehensive view of digital health literacy. Their Digital Health Literacy Instrument (DHLI) reflects this multidimensional understanding and encompasses the skills necessary for the effective use of both traditional Health 1.0 and interactive Health 2.0 technologies [5]. However, while competencies in digital literacy and health literacy are indeed interconnected with digital health literacy, it is essential to recognize that digital health literacy is based on a more intricate framework and is therefore more complex. It encompasses additional dimensions and skills that extend beyond the foundational elements of the former concepts [20]. While digital health literacy has been identified as a super social determinant of health [21], van Kessel and colleagues [20] also link the concept to civic literacy, highlighting its role in the use of digital health services in a digitally transforming society (eg, telemedicine, eHealth technologies, and digital patient records).

Although research on adult digital health literacy has been conducted in various countries [22], including Germany [23], studies specifically addressing the digital health literacy of children and adolescents remain notably scarce [14,15,24]. Current research predominantly focuses on the general health literacy of children and adolescents, examining its association with health outcomes [25] and behaviors [26,27], as well as the determinants influencing health literacy [25,28,29]. Recent findings from the German HBSC study revealed that 24.4% of 11- to 15-year-olds exhibit low health literacy, with elevated risk factors including lower family affluence, younger age, diverse gender identity, and attendance at nongrammar schools (the highest form of German secondary education) [30]. Sendatzki et al [30] reported that female adolescents and those with lower educational attainment experienced greater difficulties in managing health information, while no significant association was found for migration background. A systematic review by Fleary et al [27] identified positive associations between age and health literacy in 4 out of 8 studies [25,27], whereas a German study found that children and adolescents reported increasing difficulties in dealing with health-related information as they aged [31]. Several studies on child and adolescent health literacy have identified a social gradient in health literacy [28,31-33].

In contrast to health literacy, most existing studies on child and adolescent digital health literacy are based on selective samples, limiting the ability to draw generalizable conclusions. While research from various countries suggests that adolescents generally demonstrate a sufficient level of eHealth literacy, a significant proportion of studies still identify challenges in this area. A representative study of 1250 adolescents in Taiwan found that participants’ eHealth literacy (mean 3.69) was above average, as assessed by the eHealth Literacy Scale (eHEALS) [34]. A study involving Turkish students (N=1349) found that slightly less than half of the participants (45.6%) reported poor eHealth literacy [35]. In a recent study, Dadaczynski et al [15] examined the digital health literacy levels of eighth- and ninth-grade students in Germany, focusing on 5 dimensions: *information searching, adding self-generated content, evaluating reliability, determining relevance,* and *protecting privacy.* The findings revealed that up to 37.5% of students experienced difficulties across all digital health literacy domains, with the most frequent challenges occurring in the dimensions of *evaluating reliability* and *determining the relevance of health information* [15]. These findings align with another study in which over one-third of adolescents expressed uncertainty about fake news and misinformation [12]. Regarding sociodemographic characteristics, no differences in digital health literacy were observed based on school type or grade level [15]. By contrast, male respondents and students with high subjective social status were more likely to exhibit sufficient levels of digital health literacy. Other studies support these results, indicating that children and adolescents from less affluent families are more likely to have lower levels of health literacy [28,30], media health literacy [36], and digital health literacy [28,30]. Additionally, adolescents with moderate to low digital health literacy reported lower physical activity and higher daily consumption of fruits and soft drinks [15]. Previous studies have also identified associations between digital health literacy and more favorable health outcomes, including improved dietary practices and physical activity [35,37,38], as well as enhanced overall health status [5,37].

### Objective

Building on the existing body of research, which remains generally limited, particularly regarding representative studies, this study aims to investigate the level of digital health literacy among a representative sample of children and adolescents in Germany and its association with sociodemographic characteristics. Accordingly, this paper addresses the following research questions:

- What is the current status of digital health literacy among secondary schoolchildren in Germany?
- Are there sociodemographic differences in the digital health literacy of secondary schoolchildren in Germany?

## Methods

### Study Design and Sample

A representative cross-sectional study was conducted with children and adolescents from secondary schools in Germany between October and November 2022. To ensure the sample’s representativeness regarding age, sex, migration background, school type and form, school class, and state, data from the official school statistics report for the 2020/2021 school year, provided by the German Federal Statistical Office (Destatis), were used [39].

The recruitment of children and adolescents in Germany and data collection were conducted by an independent survey research institute, Iconkids & Youth International Research GmbH, which specializes in research involving children and adolescents. The following criteria were considered when selecting the representative sample: (1) residence in Germany and attendance at a secondary school (grades 5-10), (2) school type, (3) sex, (4) migration background, and (5) distribution across federal states and municipality size classes. To achieve this, a representative quota sample was drawn based on the criteria listed above. Participants were recruited by 375 trained youth interviewers and surveyed using computer-assisted personal interviews. Youth interviewers were selected to enhance target group engagement and leverage the benefits of peer involvement, such as increased comfort for interviewees. Following the quota system, each interviewer received specific individual instructions on the target participants to be interviewed. Interviewers recruited children and adolescents from their private social networks or through referrals from interviewees, with a maximum of 5 interviews per interviewer included in the analysis.

### Ethical Consideration

Participation in the study was voluntary, with the option to withdraw at any time, and data were collected anonymously. No incentives were provided for participation. Informed consent was obtained from both participants and their parents. The study was approved by the Ethics Committee of the Technical University of Munich, Germany (approval number 2022-401-S-NP/050, dated September 12, 2022).

### Statistical Analysis

#### Digital Health Literacy

Digital health literacy was assessed using an adapted version of the DHLI by van der Vaart and Drossaert [5]. The DHLI consists of 7 subdimensions: *operational skills, navigation skills, information searching, self-generated content, evaluating reliability, determining relevance,* and *protecting privacy.* Each subdimension includes 3 items measured on a 4-point Likert scale. The subdimensions capture competence-related challenges, such as assessing the trustworthiness of information for evaluating reliability, using appropriate keywords or search terms for information searching, and maintaining orientation on a website for navigational skills.

For all dimensions, response options ranged from “very easy,” “easy,” “difficult,” and “very difficult,” except for the dimensions protecting privacy and self-generated content, which used the options “often,” “sometimes,” “rarely,” and “never,” along with an additional option for those who do not share health-related information online. Based on cognitive interviews with young people aged 10-18 years, the DHLI was adapted to enhance item comprehension by avoiding difficult vocabulary, unclear expressions, and outdated terms [24]. Following the ITC (International Test Commission) Guidelines for Translating and Adapting Tests (2018), the adapted version [24] was translated into German and pretested with 12 children and adolescents through cognitive interviews using the think-aloud method and probing questions [40,41]. Based on the pretest results, items were refined by incorporating preferred synonyms (eg, using *handy* instead of a mobile phone), simplifying wording, and adding clarifications (eg, “information about health” instead of “health information”). In this study, the internal consistency (Cronbach α) of all 7 subscales ranged from acceptable to good (Cronbach α=0.735-0.866). An overview of the scales and items used in this article is available in Multimedia Appendix 1.

#### Sociodemographic Variables

To examine sociodemographic differences in digital health literacy, the variables sex, age, migration background, subjective family affluence, and type of school were included. Response options for biological sex were “male,” “female,” and “intersexual/intergender.” Age was assessed through an open-ended question and categorized into 3 groups for analysis: 9-11 years, 12-15 years, and 16-18 years. Following the HBSC study, migration background was classified into categories, with children and adolescents having 1 parent not born in Germany categorized as having a 1-sided history of migration. A 2-sided history of migration was recorded if the students themselves and at least one parent, or both parents, were not born in Germany [42]. Perceived family affluence was assessed using a single-item measure to examine respondents’ subjective socioeconomic status (“How would you describe the economic situation in your family?”). This item has been previously used in the HBSC study and other research on health inequalities, demonstrating good reliability and understandability for adolescents [43-45]. The response options were categorized into low family affluence (“not at all well off,” “not so well off,” and “average”) and high family affluence (“quite well off” and “very well off”) [46].

### Data Analysis

#### Score Calculation

There are currently no established cutoffs for the DHLI; therefore, a content-logically derived categorization was adopted based on the methodological approach of Dadaczynski et al [15]. A sum score was calculated for each digital health literacy dimension by summing the numerical values of all 3 items within a subscale, each ranging from 1 to 4. This resulted in a total score ranging from 3 to 12, with higher scores indicating a higher level of digital health literacy. Following Dadaczynski et al [15], the sum score was categorized into 3 groups: inadequate (3-6), representing participants who rated the items as “difficult” or “very difficult”; sufficient (9-12), for participants who rated the items as “easy” or “very easy”; and problematic (7-8), for those falling between inadequate and sufficient.

An overall score was calculated using 5 subscales of the DHLI, excluding “adding self-generated content” and “protecting privacy” due to a high number of missing values, primarily from the response option “I don’t share news related to health on the internet.” For the overall score, the categories derived from the subscale sum scores were coded as follows: 1 (inadequate), 2 (problematic), and 3 (sufficient), resulting in a total score range of 5-15. For further analyses, the overall score was categorized into 3 levels: inadequate (5-7), problematic (8-12), and sufficient digital health literacy (13-15).

#### Bivariate and Multivariate Analyses

To analyze differences between sociodemographic variables and each digital health literacy dimension, bivariate analyses were performed using cross-tabulation with subsequent *χ*^2^ tests. Effect sizes were measured using Cramér V, with thresholds defined as follows: V≥0.1 (small effect), V≥0.3 (moderate effect), and V≥0.5 (large effect) [47]. A significance level of *P*<.05 was applied for all analyses [47]. In the final step, binary logistic regression analyses were conducted to examine the association between predictor variables (sex, age, migration background, perceived family affluence, and school type) and digital health literacy dimensions. For this purpose, the digital health literacy categories “inadequate” and “problematic” were merged into a single category, “limited” digital health literacy, in alignment with the HLS-EU study [48]. Reference categories were selected based on empirical findings [15,28,30-33].

To assess potential multicollinearity among predictor variables, correlations were calculated, with *r*<0.90 indicating no multicollinearity concerns [49]. Model significance was tested using omnibus tests of model coefficients, applying a significance level of *P*<.05. Model fit was evaluated using the Hosmer-Lemeshow test, with *P*>.05 indicating a good fit. To assess the variance explained by the model, Nagelkerke *R*^2^ was calculated, with interpretation guidelines from Backhaus et al [50]: *R*^2^>0.20 (small effect), *R*^2^>0.40 (medium effect), and *R*^2^>0.50 (large effect). Additionally, the percentage of accuracy in classification was computed using a cutoff value of 0.50. Finally, odds ratios (ORs) with a 95% CI were calculated for all predictors. All statistical analyses were performed using IBM SPSS Statistics version 29.0 for Windows (IBM Corp).

## Results

### Characteristics of Participants

The final representative sample consisted of 1448 children and adolescents. Table 1 presents the sociodemographic characteristics of the study sample. Most participants were aged between 12 and 15 years and had no migration background (n=964, 66.57%). Among the 1448 children and adolescents, 824 (56.91%) rated their perceived family affluence as “average,” while only a small proportion (n=21, 1.45%) reported being “not at all well off.” Regarding school type, the largest proportion (n=515, 35.57%) attended a grammar school, followed by students in integrated comprehensive schools (n=315, 21.75%).

**Table 1:** Participant demographic data: a nationally representative cross-sectional study of secondary school students in Germany from October to November 2022.

Characteristics			Participants (N=1448), n (%)^a^
**Sex**			
	Female	712 (49.17)	
	Male	736 (50.83)	
**Age (years)**			
	9-11	389 (26.86)	
	12-15	909 (62.78)	
	16-18	150 (10.36)	
**Migration background**			
	1-sided migration background	327 (22.58)	
	2-sided migration background	157 (10.84)	
	No migration background	964 (66.57)	
**Perceived family affluence**			
	Not at all well off	21 (1.45)	
	Rather not well off	159 (10.98)	
	Average	824 (56.91)	
	Quite well off	369 (25.48)	
	Very well off	75 (5.18)	
**School type**			
	Secondary general school	116 (8.01)	
	Intermediate school	292 (20.17)	
	(Vocational) grammar school	515 (35.57)	
	Integrated comprehensive school	315 (21.75)	
	School with several levels of qualification	189 (13.05)	
	Orientation stage	20 (1.38)	
	Schools for children with special educational needs	1 (0.07)	

^a^Percentages have been rounded and may not total to 100%.

Overall, 880 out of 1362 (64.61%) children and adolescents demonstrated sufficient levels of digital health literacy, while 419 (30.76%) exhibited problematic levels, and 63 (4.63%) had inadequate levels. At the subscale level, the highest proportion of participants with limited digital health literacy (ie, problematic or inadequate) was observed in evaluating reliability (n=535, 39.28%). By contrast, the lowest proportion was found in operational skills (n=113, 8.30%).

To gain a more nuanced understanding, Table 2 lists all 21 items of the DHLI and shows the percentage of respondents who reported difficulties with each item (“difficult/very difficult” or “sometimes/often”). The 3 items that caused the least difficulty were all from the operational skills subscale. By contrast, the 3 items most frequently rated as difficult were related to protecting privacy and navigation skills (see Table 2). Difficulties with the remaining subscales ranged from 251 out of 1362 (18.43%; item 17, determining relevance) to 445 out of 1362 (32.67%; item 13, evaluating reliability).

**Table 2:** Frequency of response option “difficult/very difficult” or “sometimes/often” of the individual items of digital health literacy (n=404-1362).

Item			Values, n/N (%); 95% CI^a^
**When you search the internet for information on health, how easy or difficult is it for you to...(n=1362)**			
	1. ...use the keyboard of a computer, or a tablet, or a phone (eg, to type words)?	67 (4.92); 4.0-5.8	
	2. ...use the mouse or a touchpad (eg, to move the cursor or to click)?	74 (5.43); 4.3-6.6	
	3. ...use the buttons or links on websites?	87 (6.39); 5.1-7.5	
**When you search the internet for health information, how often does it happen that... (n=1362)**			
	4. ...you lose track of where you are on a website or the internet?	513 (37.67); 35.1-40.3	
	5. ...you don’t know how to get back to the previous page?	402 (29.52); 27.2-32.0	
	6. ...you click on something and see something that differs from what you expected?	623 (45.74); 43.2-48.2	
**When you search the internet for information on health, how easy or difficult is it for you to... (n=1362)**			
	7. ...make a choice from all the information you find?	373 (27.39); 25.0-29.7	
	8. ...use the keywords or search term to find the information you are looking for?	323 (23.72); 21.6-25.9	
	9. ...find the exact information you are looking for?	357 (26.21); 23.9-28.4	
**When sharing a message about health (eg, with your doctor; on a website, forum, blog; or on social media such as Facebook, Twitter, Snapchat, Instagram, or WhatsApp), how easy or difficult is it for you to... (n=404)**			
	10. ...clearly and precisely describe your questions on health?	94 (23.27); 19.3-27.2	
	11. ...express your opinion, thoughts, or feelings in writing?	95 (23.51); 19.6-27.2	
	12. ...write your message as such, for people to understand exactly what you mean?	98 (24.26); 20.5-28.5	
**When you search the internet for information on health, how easy or difficult is it for you to... (n=1362)**			
	13. ...decide whether the information is trustworthy or not?	445 (32.67); 30.3-34.9	
	14. ...decide whether the information is an advertisement that is trying to sell something?	345 (25.33); 23.1-27.4	
	15. ...check different online sources to see whether they provide the same information?	398 (29.22); 26.7-31.7	
**When you search the internet for information on health, how easy or difficult is it for you to... (n=1362)**			
	16. ...use the information you find to make decisions about your health (eg, on nutrition, medication or to decide whether to ask for a doctor‘s opinion)?	287 (21.07); 19.1-23.2	
	17. ...apply the information you find in your daily life (eg, physical activity, eating habits, leisure time activities)?	251 (18.43); 16.7-20.3	
	18. ...decide if the information you find relates to you, your current situation, and your life?	310 (22.76); 20.9-24.6	
**When you write a message about health on a website, in a public forum, or on social media (eg, by posting, commenting, or liking/disliking something), how often... (n=404)**			
	19. ...do you find it difficult to know who will read the message?	240 (59.41); 55.0-63.9	
	20. ...do you share your private information (eg, name or address, location, school information)?	193 (47.77); 43.3-52.5	
	21. ...do you share someone else‘s private information (eg, name or address, location, school information)?	204 (50.50); 45.5-54.2	

^a^Number of cases responded with “difficult/very difficult,” respectively, “sometimes/often” for the subscales “navigation skills” and “protecting privacy.”

### Bivariate and Multivariate Analyses

Multimedia Appendix 2 presents the differences between sociodemographic variables and DHLI dimensions. When stratified by sex, girls showed a slightly but significantly (*P*=.03) higher frequency of insufficient operational skills (11/684, 3.7%) compared with boys (25/678, 1.6%; χ22=7.14, *P*=.03). Additionally, significant age differences were observed for all DHLI dimensions except for protecting privacy (operational skills: χ24=29.99, *P*<.001; navigation skills: χ24=73.51, *P*<.001; information searching: χ24=56.98, *P*<.001; adding self-generated content: χ24=18.54, *P*=.001; evaluating reliability: χ24=65.38, *P*<.001; and determining relevance: χ24=90.52, *P*<.001). Across all 6 DHLI dimensions, children and adolescents aged 9-11 years or 12-15 years were significantly more likely to exhibit inadequate abilities compared with those aged 16-18 years (*P* value ranged from <.001 to .001; see above). When stratified by migration background, significant differences were found for navigation skills (χ24=22.13, *P*<.001) and evaluating reliability (χ24=12.87, *P*=.01). Regarding navigation skills, respondents with a 2-sided migration background more often exhibited inadequate ability (53/149, 35.6%) compared with those with a 1-sided migration background (235/910, 25.8%) or no migration background (235/910, 25.8%). By contrast, young people with a 2-sided migration background had the highest frequency of sufficient ability to evaluate the reliability of digital health information (101/149, 67.8%). Regarding school type, significant differences were found for operational skills (χ22=7.27, *P*=.03), information searching (χ22=8.14, *P*=.02), evaluating reliability (χ22=12.60, *P*=.002), and determining relevance (χ22=9.51, *P*=.009). Children and adolescents attending secondary schools other than grammar schools more frequently exhibited inadequate abilities in these DHLI dimensions (operational skills: 24/869, 2.8%; information searching: 163/869, 18.8%; determining relevance: 120/869, 13.8%; and evaluating reliability: 172/869, 19.8%) compared with those attending grammar schools. Additionally, significant differences were observed in 3 dimensions—information searching (χ22=36.78, *P*<.001), adding self-generated content (χ24=6.29, *P*=.04), and evaluating reliability (χ22=30.46, *P*<.001)—based on perceived family affluence, indicating that inadequate ability was more prevalent among those with lower perceived family affluence (see Multimedia Appendix 2).

All 7 binary logistic regression models were significant (see Tables 3 and 4); however, they explained only a small proportion of the variation in the dependent variable (*R*^2^=0.02-0.11). The overall percentage of accuracy in classification ranged from 839 out of 1362 (61.60%; navigation skills) to 1249 out of 1362 (91.70%; operational skills).

**Table 3:** Binary logistic regression analysis for limited digital health literacy skills.

Variables		Operational skills			Navigation skills			Information searching			Adding self-generated content	
		OR^a^ (95% CI)	*P* value	OR (95% CI)		*P* value	OR (95% CI)		*P* value	OR (95% CI)		*P* value
**Sex**												
	Male (reference)	1.00	—^b^	1.00		—	1.00		—	1.00		—
	Female	1.58 (1.06-2.34)	.03	1.05 (0.85-1.31)		.64	0.86 (0.68-1.09)		.21	1.01 (0.66-1.55)		.95
**Age (** **years** **)**												
	16-18 (reference)	1.00	—	1.00		—	1.00		—	1.00		—
	12-15	2.28 (0.81-6.46)	.12	1.91 (1.28-2.83)		.001	2.28 (1.42-3.70)		.001	2.65 (1.23-5.73)		.01
	9-11	5.42 (1.88-15.60)	.002	4.76 (3.06-7.39)		<.001	4.68 (2.81-7.80)		<.001	7.03 (2.73-18.09)		<.001
**Migration background**												
	Without migration background (reference)	1.00	—	1.00		—	1.00		—	1.00		—
	1-sided migration background	0.72 (0.42-1.21)	.21	0.74 (0.56-0.97)		.03	1.03 (0.78-1.36)		.84	0.95 (0.55-1.63)		.85
	2-sided migration background	1.22 (0.69-2.15)	.50	1.14 (0.80-1.63)		.47	0.62 (0.42-0.93)		.02	0.30 (0.13-0.66)		.003
**Perceived family affluence**												
	High (reference)	1.00	—	1.00		—	1.00		—	1.00		—
	Low	1.06 (0.68-1.65)	.79	1.01 (0.80-1.29)		.92	2.18 (1.66-2.87)		<.001	1.77 (1.13-2.76)		.01
**School**												
	Grammar school (reference)	1.00	—	1.00		—	1.00		—	1.00		—
	All other school types	1.38 (0.88-2.18)	.17	0.97 (0.77-1.24)		.82	1.04 (0.80-1.34)		.78	1.01(0.65-1.58)		.96
	*R* ^2^	0.063	—	0.075		—	0.094		—	0.111		—

^a^OR: odds ratio.

^b^Not applicable.

**Table 4:** Additional binary logistic regression analysis for limited digital health literacy skills.

Variables			Evaluating reliability				Determining relevance					Protecting privacy		
			OR^a^ (95% CI)		*P* value		OR (95% CI)		*P* value		OR (95% CI)			*P* value
**Sex**														
	Male (reference)	1.00		—^b^		1.00		—		1.00			—	
	Female	1.03 (0.82-1.29)		.08		0.99 (0.78-1.26)		.95		0.89 (0.60-1.35)			.59	
**Age (** **years** **)**														
	16-18 (reference)	1.00		—		1.00		—		1.00			—	
	12-15	1.78 (1.17-2.72)		.007		1.70 (1.05-2.74)		.03		1.14 (0.62-2.10)			.68	
	9-11	3.82 (2.41-6.04)		<.001		4.76 (2.86-7.92)		<.001		0.99 (0.44-2.23)			.99	
**Migration background**														
	Without migration background (reference)	1.00		—		1.00		—		1.00			—	
	1-sided migration background	1.19 (0.91-1.56)		.21		1.03 (0.77-1.38)		.85		1.45 (0.84-2.49)			.19	
	2-sided migration background	0.66 (0.45-0.96)		.03		0.74 (0.49-1.11)		.14		1.20 (0.62-2.32)			.58	
**Perceived family affluence**														
	High (reference)	1.00		—		1.00		—		1.00			—	
	Low	1.67 (1.3-2.15)		<.001		1.58 (1.20-2.08)		<.001		1.31 (0.86-2.01)			.21	
**School**														
	Grammar school (reference)	1.00		—		1.00		—		1.00			—	
	All other school types	1.12 (0.88-1.43)		.36		1.06 (0.81-1.37)		.69		1.19 (0.77-1.83)			.43	
	*R* ^2^	0.077		—		0.090		—		0.019			—	

^a^OR: odds ratio.

^b^Not applicable.

Regarding operational skills, significant associations were found only for sex and age: being female was associated with a 1.6-fold increased risk (OR 1.58, *P*=.03) of having limited operational skills, while belonging to the 9- to 11-year age group was associated with a 5.4-fold increased risk (OR 5.42, *P*=.002). Concerning navigation skills, being aged 9-11 years (OR 4.76, *P*<.001) or 12-15 years (OR 1.91, *P*=.001) significantly increased the likelihood of having limited navigation skills, whereas a 1-sided migration background (OR 0.74, *P*=.03) was associated with a decreased likelihood.

For information searching and evaluating reliability, being aged 9-11 years (OR_information searching_ 4.68, *P*<.001 and OR_evaluating reliability_ 3.82, *P*<.001) or 12-15 years (OR_information searching_ 2.28, *P*=.001 and OR_evaluating reliability_ 1.78, *P*=.007) and having low perceived family affluence (OR_information searching_ 2.18, *P*<.001 and OR_evaluating reliability_ 1.67, *P*<.001) increased the likelihood of having limited skills, respectively. By contrast, a 2-sided migration background reduced the likelihood of limited skills for information searching (OR 0.62, *P*=.02) and evaluating reliability (OR 0.66, *P*=.03).

For self-generated content, belonging to the age groups of 9-11 years (OR 7.03, *P*<.001) or 12-15 years (OR 2.66, *P*=.01) and having a low subjective family affluence (OR 1.77, *P*=.01) were significant predictors of limited skills. Conversely, having a 2-sided migration background (OR 0.30, *P*=.003) increased the likelihood of sufficient skills in self-generated content. Furthermore, being aged 9-11 years (OR 4.76, *P*<.001) or 12-15 years (OR 1.67, *P*=.03) and having a low perceived family affluence (OR 1.58, *P*<.001) were significant predictors of limited skills in determining relevance. By contrast, school type was not a significant factor in any binary logistic regression model (operational skills: *P*=.17; navigation skills: *P*=.82; information searching: *P*=.78; self-generated content: *P*=.96; evaluating reliability: *P*=.36; determining relevance: *P*=.70; and protecting privacy: *P*=.43).

## Discussion

### Digital Health Literacy

This cross-sectional study is the first representative study in Germany to explore digital health literacy among children and adolescents aged 9-18 and its associations with sociodemographic characteristics. Overall, the findings indicate that more than one-third of participants faced difficulties with digital health literacy (problematic 419/1362, 30.76%; inadequate 63/1362, 4.63%). While research exists on related concepts such as eHealth literacy, studies specifically examining digital health literacy in children and adolescents using the DHLI remain limited. A prior representative study on eHealth literacy among adolescents in Taiwan found that participants’ eHealth literacy (mean 3.69) was above average, as assessed using an adapted short form of the eHEALS. Comparing our findings with eHealth literacy studies, research among Turkish students (N=1349) [35] and Taiwanese students (N=1250) [34] shows similar results. Taiwanese students demonstrated a mean eHEALS score above average (mean 3.69), while the majority of Turkish students exhibited adequate eHealth literacy skills (54.4%), though a considerable proportion had limited skills (45.6%) [35]. Although health literacy is a broader and more general construct, findings from the HBSC study across 10 European countries align with our results. The study highlights children’s and adolescents’ challenges in managing health information [25,35], with more than two-thirds of school-aged children (67.2%) exhibiting only moderate levels of health literacy.

### Dimensions of Digital Health Literacy

At the subscale level, the greatest difficulties were observed in protecting privacy and navigation skills, while the least difficulties were found in operational skills and determining relevance. These results were partially consistent with findings from a previous study in Germany [15], which used 5 DHLI subscales, except for determining the relevance of online health information. Our study confirmed the previous study’s assumption that operational skills are less relevant for adolescents, as they did not report significant difficulties in this dimension. In our study, children and adolescents reported the least difficulties with the most technically related navigation skill—returning to the previous page when browsing websites. However, 623 out of 1362 (45.74%) participants occasionally or frequently encountered content that diverged from their expectations, and 513 out of 1362 (37.67%) reported losing their sense of navigation on websites or the broader internet. Given the vast amount of health information, including misinformation, that children and adolescents are exposed to [9,12], it is reasonable to conclude that encountering unexpected content is both challenging and, to some extent, unavoidable, even when a website’s headline aligns with their search query.

In contrast to our findings, Dadaczynski et al [15] reported different results when examining the subscales, with participants experiencing the greatest challenges in evaluating information reliability and determining relevance, categorizing these tasks as difficult or very difficult. However, at the item level, both studies aligned in observing that participants faced the most significant difficulties in understanding the potential readership of their messages, particularly concerning privacy protection. Given that the majority of participants (958/1448, 66.16%) in our study reported refraining from sharing health-related news online, it is important to examine whether this behavior reflects a broader trend among children and adolescents. Additionally, this raises the question of whether their infrequent engagement in sharing health-related content correlates with—or even contributes to—the observed limitations in privacy protection skills within this demographic. This finding underscores the need for further research into the relationship between health information–sharing behaviors and the development of privacy management skills among young individuals in digital environments. This interpretation aligns with existing literature suggesting that students often demonstrate inconsistent and inadequate application of data protection and privacy measures [51]. Notably, a German study found that approximately half of participants aged 14-24 years expressed uncertainty about their ability to effectively safeguard their online data. Additionally, Stoilova et al [52] suggested that children’s and adolescents’ capacities for managing data protection and privacy are limited, as their understanding of privacy is primarily interpersonal and lacks awareness of institutional and commercial contexts—posing significant risks to their data security [52]. Another possible explanation for adolescents’ reluctance to share health information online is their tendency to equate health with disease. As a result, they may not perceive themselves as sharing health-related content, even when posting about broader aspects of well-being. Future studies should further explore this perspective.

In the dimension of reliability assessment, children and adolescents faced the greatest challenges in evaluating the credibility of health information (item 13), with 445 out of 1362 (32.67%) reporting difficulties. This finding is consistent with the observations of Dadaczynski et al [15], highlighting a persistent struggle among this population in assessing the trustworthiness of health information sources. Differences emerged in the item assessing the comparison of diverse sources for informational congruence (item 15). Our findings indicate that 398 out of 1362 (29.22%) participants experienced difficulties in this area, contrasting with the 15.3% reported in the aforementioned study [15]. Adolescents use cross-referencing as a strategy to assess the credibility of health information and claims [53]. In their systematic review, Freeman et al [54] identified additional criteria adolescents consider when evaluating online health information beyond cross-checking sources. These include the website’s name and reputation, the first impression of the website, and the content it presents. These findings highlight the multifaceted approach adolescents take in assessing the credibility and reliability of health-related information encountered online. The inclusion of factors such as website reputation and first impressions underscores the crucial role of both established credibility and immediate user experience in shaping adolescents’ perceptions of digital health sources. Given the evidence on adolescents’ perceptions of misinformation and disinformation [53,55,56], along with the overwhelming amount of health information they encounter [9,12], it is plausible that these factors contribute to their difficulties in critically evaluating health content. This intersection of awareness and information overload may further complicate adolescents’ health information appraisal processes and should be considered when designing interventions.

### Sociodemographic Differences

This study found no statistically significant associations between sex and any dimensions of digital health literacy, except for operational skills. This aligns with the findings of 2 prior studies on general health literacy conducted in Germany [28,57]. Similarly, previous research on eHealth literacy using the eHEALS instrument reported no association between sex and eHealth literacy [58,59]. However, the most comparable study to ours identified correlations between sex and digital health literacy in the dimensions of information searching, adding self-generated content, and evaluating reliability, with male participants demonstrating higher abilities in these areas [15]. By contrast, some studies have identified an association between sex and digital health literacy [36], as well as health literacy [25], indicating higher health literacy among girls. The existing literature on sex-based disparities in digital health literacy presents a complex and heterogeneous landscape, making it difficult to draw definitive generalizations. This ambiguity may largely stem from the diverse measurement instruments used across studies, leading to inconsistent and sometimes contradictory findings. As a result, determining sex differences in digital health literacy remains challenging, highlighting the need for further research with standardized methodologies to better understand potential disparities.

Age was a predictor for all dimensions of digital health literacy except for protecting privacy, suggesting that older adolescents (16-18 years) have greater proficiency in managing digital health information. This finding is plausible, as it aligns with established patterns indicating that cognitive and literacy skills typically improve with age and maturation [60]. Moreover, findings from the HBSC study have consistently shown a higher prevalence of limited health literacy among younger demographics [25,30]. It is also possible that younger individuals struggle to fully comprehend survey items, leading to inaccurate responses [30]. However, a study of fourth graders in Germany found a high level of health literacy, suggesting that primary schoolchildren do not face major difficulties in this area [28]. Given the lack of significant correlations in other studies, such as those conducted by Dadaczynski et al [15] and a Turkish study on schoolchildren, the existing literature presents mixed findings regarding the relationship between digital health literacy and age. As noted earlier, the substantial proportion of respondents (958/1448, 66.16%) who reported not sharing health-related content online may indicate a general lack of experience across all age groups in safeguarding health-related privacy. Additionally, our findings reveal a positive association between digital health literacy and family affluence, consistent with previous research on health literacy [28,30], media health literacy [36], and digital health literacy [15]. While not all dimensions showed a significant association with perceived family affluence, our findings highlighted notable correlations in evaluating information reliability and determining relevance. This suggests a potential socioeconomic influence on these specific aspects of information assessment, as observed in numerous other studies. Additionally, the HBSC study, which identified social inequalities in adolescents’ social media use—where those from higher-affluence families were more likely to engage in online interactions with their social environment—supports our findings [61]. Furthermore, children and adolescents with a 2-sided migration background demonstrated higher skills in information searching, adding self-generated content, and evaluating reliability, while those with a 1-sided migration background exhibited better navigation skills. Findings from previous studies on children and adolescents have been rather heterogeneous. The German HLS-MIG study [62] found that participants with a migration background exhibited higher health literacy and digital health literacy levels. However, a study on fourth graders in Germany did not observe any significant differences based on migration background [32]. By contrast, research on 18- to 25-year-olds indicated lower health literacy levels among those with a migration background [57]. Additionally, the International Computer and Information Literacy Study (ICILS) reported that young people without a migration background demonstrated significantly higher computer and information-related skills compared with those with a migration background [63]. By contrast, a plausible explanation for this outcome is that children and adolescents with a migration background may develop greater proficiency in using digital platforms for information acquisition. This could stem from increased reliance on online resources due to parental linguistic challenges or limited literacy in the host country’s language. Consequently, they may cultivate stronger digital (health) literacy skills compared with their nonmigrant peers. However, the indicator used in this study—distinguishing between 1- and 2-sided migration backgrounds—does not fully capture the complexity of migration-related factors. It does not account for variables such as duration of residence in Germany or parental language use, making direct comparisons with existing literature challenging. Therefore, these results and hypotheses should be interpreted with caution. Further qualitative studies are needed to provide in-depth explanations of the challenges in evaluating the reliability of health information.

School type was not associated with any dimension of digital health literacy. Similar findings were observed in another study on school-aged children in Germany [15], supporting the validity of our results. Although an educational gradient in adolescents’ media health literacy was observed in an Israeli study [36], comparability to German studies remains questionable due to differences in educational systems, temporal contexts, and methodological instruments. There appears to be a reciprocal relationship between health literacy and academic performance. Several studies indicate that schoolchildren with lower health literacy tend to struggle more in school and achieve less academically [30,64]. Conversely, a Lithuanian study [65] found higher academic achievement to be a strong indicator of improved health literacy skills. This suggests that enhancing health literacy may benefit educational outcomes and academic achievement, while academic success could, in turn, contribute to better health literacy [66]. Further research is needed to determine whether this relationship also applies to digital health literacy.

### Strengths and Limitations

Our study has several strengths and limitations. It provides the first representative findings on children and adolescents’ digital health literacy in Germany and internationally. The representative nature of our sample enhances the validity of extrapolating these findings to the broader population of children and adolescents in Germany. However, whether these results apply in an international context remains to be determined. Additionally, involving young people as interviewers can increase interviewees’ comfort, potentially leading to greater truthfulness in their responses. However, the study has several limitations. First, social desirability bias cannot be ruled out. To mitigate this, youth interviewers were employed to enhance interviewees’ comfort. Second, regarding the instrument, studies using the DHLI in children and adolescents are limited. Additionally, various methods exist for calculating the DHLI sum score, making comparisons with other studies challenging. Furthermore, research on child and adolescent digital health literacy remains scarce. A systematic review by Lee et al [67] has already highlighted the methodological heterogeneity in assessing eHealth literacy and digital health literacy, emphasizing the lack of standardization in measurement approaches. The authors argue that while the eHEALS instrument is widely used, its content requires revision to align with contemporary technological advancements [67], a need previously highlighted by van der Vaart and Drossaert [5]. In response to this criticism, Le et al [68] recently introduced the HLS_19_-DIGI scale, validating its 8-item structure using Rasch modeling. To maintain comparability with a prior study in Germany [15], this study applied the same score calculation methodology. Third, a considerable number of participants in our study were coded as missing in the subscales for self-generated content and protecting privacy, as they indicated that they would not “write a message on health on the internet.” This suggests that the results for these subscales may reflect a higher proportion of children and adolescents with sufficient skills. However, those who do not write health-related messages may have been excluded from the findings, despite potentially facing challenges due to a lack of practice. Therefore, further research should reassess the suitability of this DHLI subscale when applying it to children and adolescents. Fourth, the DHLI, like other digital health literacy measurement tools, relies on self-reporting, which may introduce bias and lead to discrepancies between reported and actual digital health literacy skills. While objective methods for measuring health literacy in adolescents exist [69], comparable approaches for assessing digital health literacy remain limited.

Fifth, regarding the statistical approach, the examined predictors explained only a limited proportion of the variance. Although all models were statistically significant, the *R*^2^ values remained low (*R*^2^=0.019-0.111), indicating a small effect. Given the inherent challenges in predicting human behavior within the social sciences, further research is needed to explore additional variables that could enhance the predictive power of these models.

Sixth, the cross-sectional nature of this study limits the ability to establish causal relationships, necessitating a cautious interpretation of the reported associations. The findings provide only a snapshot at a single point in time rather than capturing a longitudinal progression of cause and effect.

### Recommendations

Integrating (digital) health literacy into the school curriculum and enhancing schools’ organizational health literacy are among the most promising strategies for sustainably promoting health literacy in educational settings [70]. At the behavioral level, interventions for teachers and parents—such as Massive Open Online Courses and additional training programs—are essential. For schoolchildren, embedding digital health literacy into existing curricula, including mandatory digital and media literacy programs or health education curricula, is a crucial approach. While initial efforts have been made, more systematic implementation is needed [71]. Although further research is required to assess the long-term health effects of improving digital health literacy in children and adolescents, it has already been recognized as a key determinant of health. Therefore, it demands greater attention as a critical health policy priority in Germany and beyond, ensuring sustained investment and funding for its development from an early stage in schools.

### Conclusions

This study makes a valuable contribution to the existing literature and helps bridge the evidence gap in child and adolescent digital health literacy and its associated sociodemographic factors. Our findings indicate a significant prevalence of low digital health literacy among children and adolescents in Germany, with 419 out of 1362 (30.76%) classified as having problematic levels and 63 out of 1362 (4.63%) as inadequate. Furthermore, sociodemographic variables were linked to all dimensions of digital health literacy, suggesting a social gradient in digital health literacy among this population. These findings highlight the urgent need for targeted interventions to improve digital health literacy among children and adolescents, equipping them with the necessary skills to navigate complex information environments in an increasingly digitalized society. Schools play a crucial role in fostering digital health literacy among school-aged children. Given our findings on significant social disparities, special attention should be directed toward children and adolescents from less affluent families. In this context, interventions should be designed at multiple levels: at the behavioral level, by strengthening personal capacities and agency, and at the environmental level, by enhancing school development, reducing environmental complexities, and improving setting-related structures [72].

## Appendix

Multimedia Appendix 1 Overview of scales and items.

Multimedia Appendix 2 Digital health literacy overall and stratified by sociodemographic and school-related factors.

## Abbreviations

DHLI: Digital Health Literacy Instrument
eHEALS: eHealth Literacy Scale
ICILS: International Computer and Information Literacy Study
ITC: International Test Commission
OR: odds ratio

## Data Availability

The data that support the findings of this study are available from the corresponding author upon reasonable request.
