# Supplementary material for: Digital Health Literacy of Children and Adolescents and Its Association With Sociodemographic Factors: Representative Study Findings From Germany
Source: J Med Internet Res. 2025 May 5;27:e69170. doi: 10.2196/69170 (PMC12089873; doi:10.2196/69170)
Supplement: Multimedia Appendix 1 [file jmir_v27i1e69170_app1.docx]

**Multimedia Appendix** Overview of scales and items**.**

## This appendix contains all items and scales that are used for this paper

## Sociodemographic information and school characteristic

1. Sex
2. Age
3. Migration background participant
4. Migration background mother
5. Migration background father
6. School type
7. Family affluance

## Digital health literacy

1. Operational skills
2. Information searching
3. Self-generated content
4. Navigation skills
5. Protecting privacy
6. Determining relevance
7. Evaluating reliability

## Sociodemographic information and school characteristic

| a | Please indicate your biological sex. Are you ... | | | | | |  |
| --- | --- | --- | --- | --- | --- | --- | --- |
|  | 🞏 | Male | 🞏 | Female | 🞏 | Intersexual | |

| b | How old are you? |  |
| --- | --- | --- |
|  | I am _____ years old. | |

| c | In what country were you born? | | | | | |  |
| --- | --- | --- | --- | --- | --- | --- | --- |
|  | 🞏 | Germany | 🞏 | Other country, namely ____________ | 🞏 | Not specified | |

| d | In what country was your mother born? | | | | | |  |
| --- | --- | --- | --- | --- | --- | --- | --- |
|  | 🞏 | Germany | 🞏 | Other country, namely ____________ | 🞏 | Not specified | |

| e | In what country was your father born? | | | | | |  |
| --- | --- | --- | --- | --- | --- | --- | --- |
|  | 🞏 | Germany | 🞏 | Other country, namely ____________ | 🞏 | Not specified | |

| f | What kind of school are you attending in the current school year? | |  |
| --- | --- | --- | --- |
|  | Primary School | 🞏 | |
|  | Secondary school (Hauptschule) | 🞏 | |
|  | Secondary school (Realschule) | 🞏 | |
|  | Secondary school (Gymnasium) | 🞏 | |
|  | Cooperative secondary school / Integrated secondary school^1^ | 🞏 | |
|  | School with several educational programs (e.g., district or comprehensive school) | 🞏 | |
|  | Schools for children with special educational needs* | 🞏 | |
|  | Other type of school, namely | _______ | |

| g | How wealthy do you consider your family to be? "Very wealthy" refers to families with the most money. "Not at all wealthy" means the families with the least money. Here is a scale you can use to grade your answer. Please click on the answer that fits your family best. | | | | |  |
| --- | --- | --- | --- | --- | --- | --- |
|  | Very wealthy | Rather wealthy | Average | Rather not wealthy | Not wealthy at all | |
|  | 🞏 | 🞏 | 🞏 | 🞏 | 🞏 | |

## Digital health literacy

|  | We know from interviews with other children and adolescents that for some it is very easy to find and understand the appropriate information on health on the internet. Other children and adolescents have difficulty finding information on this on the internet, or they can use the information they find. This is exactly what the next questions are about: I would like to know how easy or difficult it is for you personally to use the internet as a source of information on health topics. |
| --- | --- |

| a | When you search the internet for information on health, how easy or difficult is it for you to… | | | | | |  |
| --- | --- | --- | --- | --- | --- | --- | --- |
|  |  | | Very easy | Easy | Difficult | Very difficult | |
|  | ... | use the keyboard of a computer, or a tablet or a phone (e.g., to type words)? (invert) | 🞏 | 🞏 | 🞏 | 🞏 | |
|  | ... | use the mouse or a touchpad (e.g., to move the cursor or to click)? (invert) | 🞏 | 🞏 | 🞏 | 🞏 | |
|  | … | use the buttons or links on websites? (invert) | 🞏 | 🞏 | 🞏 | 🞏 | |

| b | If you don't share news related to health on the Internet, pick the last response option and move to the next question.  When sharing a message about health (e.g., to your doctor; on a website; in a forum; on a blog, or on social media such as Facebook, Twitter, Snapchat, Instagram, or WhatsApp), how easy or difficult is it for you to ... | | | | | |  |
| --- | --- | --- | --- | --- | --- | --- | --- |
|  |  | | Very easy | Easy | Difficult | Very difficult | |
|  | ... | clearly and precisely describe your questions on health? (invert) | 🞏 | 🞏 | 🞏 | 🞏 | |
|  | ... | express your opinion, thoughts, or feelings in writing? (invert) | 🞏 | 🞏 | 🞏 | 🞏 | |
|  | … | write your message as such, for people to understand exactly what you mean? (invert) | 🞏 | 🞏 | 🞏 | 🞏 | |
|  |  | I don't share news related to health on the Internet | 🞏 | | | | |

| c | When you search the internet for health information, how often does it happen that ... | | | | | |  |
| --- | --- | --- | --- | --- | --- | --- | --- |
|  |  | | Often | Some- times | Rarely | Never | |
|  | ... | you lose track of where you are on a website or the internet? | 🞏 | 🞏 | 🞏 | 🞏 | |
|  | ... | you don’t know how to get back to the previous page? | 🞏 | 🞏 | 🞏 | 🞏 | |
|  | … | you click on something and see something that differs from what you expected? | 🞏 | 🞏 | 🞏 | 🞏 | |

| d | If you don't share news related to health on the Internet, pick the last response option and move to the next question.  When you write a message about health on a website, in a public forum, or social media (e.g., post, comment, or like/dislike something), how often... | | | | | |  |
| --- | --- | --- | --- | --- | --- | --- | --- |
|  |  | | Often | Some-times | Rarely | Never | |
|  | ... | do you find it difficult to know who will read the message? | 🞏 | 🞏 | 🞏 | 🞏 | |
|  | ... | do you share your private information (e.g., name or address, location, school information)? | 🞏 | 🞏 | 🞏 | 🞏 | |
|  | … | do you share someone else’s private information (e.g., name or address, location, school information)? | 🞏 | 🞏 | 🞏 | 🞏 | |
|  |  | I don't share news related to health on the Internet | 🞏 | | | | |

| e | When you search the internet for information on health, how easy or difficult is it for you to ... | | | | | |  |
| --- | --- | --- | --- | --- | --- | --- | --- |
|  |  | | Very easy | Easy | Difficult | Very difficult | |
|  | ... | make a choice from all the information you find? (invert) | 🞏 | 🞏 | 🞏 | 🞏 | |
|  | ... | use the key words or search term to find the information you are looking for? (invert) | 🞏 | 🞏 | 🞏 | 🞏 | |
|  | … | find the exact information you are looking for? (invert) | 🞏 | 🞏 | 🞏 | 🞏 | |

| f | When you search the internet for information on health, how easy or difficult is it for you to... | | | | | |  |
| --- | --- | --- | --- | --- | --- | --- | --- |
|  |  | | Very easy | Easy | Difficult | Very difficult | |
|  | ... | use the information you find to make decisions about your health (e.g. on nutrition, medication or to decide whether to ask for a doctor’s opinion)? (invert) | 🞏 | 🞏 | 🞏 | 🞏 | |
|  | ... | apply the information you find in your daily life (e.g., physical activity, eating habits, leisure time activities)? (invert) | 🞏 | 🞏 | 🞏 | 🞏 | |
|  | … | decide if the information you find relates to you, your current situation and your life? (invert) | 🞏 | 🞏 | 🞏 | 🞏 | |

| g | When you search the internet for information on health, how easy or difficult is it for you to... | | | | | |  |
| --- | --- | --- | --- | --- | --- | --- | --- |
|  |  | | Very easy | Easy | Difficult | Very difficult | |
|  | ... | decide whether the information is trustworthy or not? (invert) | 🞏 | 🞏 | 🞏 | 🞏 | |
|  | ... | decide whether the information is an advertisement that is trying to sell something? (invert) | 🞏 | 🞏 | 🞏 | 🞏 | |
|  | … | check different online sources to see whether they provide the same information? (invert) | 🞏 | 🞏 | 🞏 | 🞏 | |
