# Supplementary material for: Digital Health Literacy of Children and Adolescents and Its Association With Sociodemographic Factors: Representative Study Findings From Germany
Source: J Med Internet Res. 2025 May 5;27:e69170. doi: 10.2196/69170 (PMC12089873; doi:10.2196/69170)
Supplement: Multimedia Appendix 2 [file jmir_v27i1e69170_app2.docx]

|  |  | **Operational skills^a^** | | | **Navigation skills^a^** | | | **Information searching^a^** | | | **Adding self-generated content^a^** | | |
| --- | --- | --- | --- | --- | --- | --- | --- | --- | --- | --- | --- | --- | --- |
|  | | *Sufficient* | *Problematic* | *Inadequate* | *Sufficient* | *Problematic* | *Inadequate* | *Sufficient* | *Problematic* | *Inadequate* | *Sufficient* | *Problematic* | *Inadequate* |
| **%, n** | | 91.7 (1249) | 5.7 (77) | 2.6 (36) | 54.8 (646) | 20.4 (278) | 24.7 (337) | 66.2 (902) | 16.9 (230) | 16.9 (230) | 63.6 (257) | 25.2 (102) | 11.1 (45) |
| **Sex** | | χ^2^_2_=7.14*, P*=.028, V=.072 | | | n.s.^b^ | | | n.s. | | | n.s. | | |
|  | Male %, (n) | 93.4 (639) | 5.0 (34) | 1.6 (11) | 55.7 (381) | 21.3 (146) | 23.0 (157) | 64.8 (443) | 17.5 (120) | 17.7 (121) | 64.0 (119) | 26.3 (49) | 9.7 (18) |
|  | Female %, (n) | 90.0 (610) | 6.3 (43) | 3.7 (25) | 54.0 (366) | 19.5 (132) | 26.5 (180) | 67.7 (459) | 16.2 (110) | 16.1 (109) | 63.3 (138) | 24.3 (53) | 12.4 (27) |
| **Age** | | χ^2^_4_=29.99*, P*<.001, V=.105 | | | χ^2^_4_=73.51*, P*<.001, V=.164 | | | χ^2^_4_=56.98*, P*<.001, V=.145 | | | χ^2^_4_=18.54*, P*=.001, V=.151 | | |
|  | 9-11 %, (n) | 84.9 (287) | 10.4 (35) | 4.7 (16) | 36.4 (123) | 30.8 (104) | 32.8 (111) | 52.4 (177) | 21.0 (71) | 26.6 (90) | 43.8 (21) | 41.7 (20) | 14.6 (7) |
|  | 12-15 %, (n) | 93.4 (819) | 4.6 (40) | 2.1 (18) | 59.0 (517) | 18.1 (159) | 22.9 (201) | 68.5 (601) | 16.8 (147) | 14.7 (129) | 63.1 (190) | 25.6 (77) | 11.3 (34) |
|  | 16-18 %, (n) | 97.3 (143) | 1.4 (2) | 1.4 (2) | 72.8 (107) | 10.2 (15) | 17.0 (25) | 84.4 (124) | 8.2 (12) | 7.5 (11) | 83.6 (46) | 9.1 (5) | 7.3 (4) |
| **Migration background** | | n.s. | | | χ^2^_4_=22.13*, P*<.001, V =.09 | | | n.s. | | | n.s. | | |
|  | One-sided %, (n) | 93.7 (284) | 4.6 (14) | 1.7 (5) | 60.7 (184) | 23.1 (70) | 16.2 (49) | 64.4 (195) | 16.5 (50) | 19.1 (58) | 64.6 (51) | 26.6 (21) | 8.9 (7) |
|  | Two-sided %, (n) | 88.6 (132) | 9.4 (14) | 2.0 (3) | 48.3 (72) | 16.1 (24) | 35.6 (53) | 73.2 (109) | 15.4 (23) | 11.4 (17) | 81.3 (39) | 14.6 (7) | 4.2 (2) |
|  | Without %, (n) | 91.5 (833) | 5.4 (49) | 3.1 (28) | 54.0 (491) | 20.2 (184) | 25.8 (235) | 65.7 (598) | 17.3 (157) | 17.0 (155) | 60.3 (167) | 26.7 (74) | 13.0 (36) |
| **School** | | χ^2^_2_=7.27*, P*=.026, V=.073 | | | n.s. | | | χ^2^_2_=8.14*, P*=.017, V=.077 | | | n.s. | | |
|  | (Vocational) grammar school %, (n) | 94.1 (464) | 3.4 (17) | 2.4 (12) | 57.8 (285) | 19.7 (97) | 22.5 (111) | 70.8 (349) | 15.6 (77) | 13.6 (67) | 66.5 (115) | 23.1 (40) | 10.4 (18) |
|  | All other schools %, (n) | 90.3 (785) | 6.9 (60) | 2.8 (24) | 53.2 (462) | 20.8 (181) | 26.0 (226) | 63.6 (553) | 17.6 (153) | 18.8 (163) | 615 (142) | 26.8 (62) | 11.7 (27) |
| **Perceived family affluence** | |  | n.s. |  | n.s. | | | χ^2^_2_=36.78*, P*<.001, V=.164 | | | χ^2^_4_=6.29*, P*=.043, V=.125 | | |
|  | Not at all-/ not so well off/ average %, (n) | 91.4 (867) | 5.9 (56) | 2.7 (26) | 54.4 (516) | 21.7 (206) | 23.9 (227) | 61.1 (580) | 19.2 (182) | 19.7 (187) | 58.6 (133) | 27.8 (63) | 13.7 (31) |
|  | Quite well off/very well off %, (n) | 92.5 (382) | 5.1 (21) | 2.4 (10) | 55.9 (231) | 17.4 (72) | 26.6 (110) | 78.0 (322) | 11.6 (48) | 10.4 (43) | 70.1 (124) | 22.0 (39) | 7.9 (14) |
| **Total** | | 91.7 (1249) | 5.7 (77) | 2.6 (36) | 54.8 (747) | 20.4 (278) | 24.7 (337) | 66.2 (902) | 16.9 (230) | 16.9 (230) | 63.6 (257) | 25.2 (102) | 11.1 (45) |

Table 3. (Continued)

|  |  | **Evaluating reliability^a^** | | | **Determining relevance^a^** | | | **Protecting privacy/ Data protection^a^** | | |
| --- | --- | --- | --- | --- | --- | --- | --- | --- | --- | --- |
|  | | *Sufficient* | *Problematic* | *Inadequate* | *Sufficient* | *Problematic* | *Inadequate* | *Sufficient* | *Problematic* | *Inadequate* |
| **%, n** | | 36.1 (146) | 24.8 (100) | 39.1 (158) | 70.1 (955) | 18.0 (245) | 11.9 (162) | 60.7 (827) | 22.0 (300) | 17.3 (235) |
| **Sex** | | n.s. | | | n.s. | | | n.s. | | |
|  | Male %, (n) | 61.1 (418) | 21.6 (148) | 17.3 (118) | 70.2 (480) | 19.2 (131) | 10.7 (73) | 34.9 (65) | 28.0 (52) | 37.1 (69) |
|  | Female %, (n) | 60.3 (409) | 22.4 (152) | 17.3 (117) | 70.1 (475) | 16.8 (114) | 13.1 (162) | 37.2 (81) | 22.0 (48) | 40.8 (89) |
| **Age** | | χ^2^_4_=65.38*, P*<.001, V=.155 | | | χ^2^_4_=90.52*, P*<.001, V=.182 | | | n.s. | | |
|  | 9-11 %, (n) | 45.6 (154) | 25.4 (86) | 29.0 (98) | 51.8 (175) | 24.9 (84) | 23.4 (79) | 39.6 (19) | 31.3 (15) | 29.2 (14) |
|  | 12-15 %, (n) | 63.7 (559) | 22.0 (193) | 14.3 (125) | 74.8 (656) | 16.1 (141) | 9.1 (80) | 34.9 (105) | 25.2 (76) | 39.9 (120) |
|  | 16-18 %, (n) | 77.6 (114) | 14.3 (21) | 8.2 (12) | 84.4 (124) | 13.6 (10) | 2.0 (3) | 40.4 (22) | 16.4 (9) | 43.6 (24) |
| **Migration background** | | χ^2^_2_=12.87*, P*=.012, V=.07 | | | n.s. | | | n.s. | | |
|  | One-sided %, (n) | 56.4 (171) | 28.7 (87) | 14.9 (45) | 69. (209) | 20.1 (61) | 10.9 (33) | 30.4 (24) | 32.9 (26) | 36.7 (29) |
|  | Two-sided %, (n) | 67.8 (101) | 16.8 (25) | 15.4 (23) | 73.2 (109) | 14.8 (22) | 12.1 (18) | 33.3 (16) | 18.8 (9) | 47.9 (23) |
|  | Without %, (n) | 61.0 (555) | 20.7 (188) | 18.4 (167) | 70.0 (637) | 17.8 (162) | 12.2 (111) | 38.3 (106) | 23.5 (65) | 38.3 (106) |
| **School** | | χ^2^_2_=12.60*, P*=.002, V=.096 | | | χ^2^_2_=9.51*, P*=.009, V=.084 | | | n.s. | | |
|  | (Vocational) grammar school %, (n) | 65.9 (325) | 21.3 (105) | 12.8 (63) | 74.2 (366) | 17.2 (85) | 8.5 (42) | 39.3 (68) | 23.7 (41) | 37.0 (64) |
|  | All other schools %, (n) | 57.8 (502) | 22.4 (195) | 19.8 (172) | 67.8 (589) | 18.4 (160) | 13.8 (120) | 33.8 (78) | 25.5 (59) | 40.7 (94) |
| **Perceived family affluence** | | χ^2^_2_=30.46*, P*<.001, V=.150 | | | χ^2^_2_=15.03*, P*<.001, V=.105 | | | n.s. | | |
|  | Not at all-/ not so well off/ average %, (n) | 56.8 (539) | 22.4 (213) | 20.8 (197) | 67.1 (637) | 19.2 (182) | 13.7 (130) | 33.0 (75) | 25.1 (57) | 41.9 (95) |
|  | Quite well off/very well off %, (n) | 69.7 (288) | 21.1 (87) | 9.2 (38) | 77.0 (318) | 15.3 (63) | 7.7 (32) | 40.1 (71) | 24.3 (43) | 35.6 (63) |
| **Total** | | 60.7 (827) | 22.0 (300) | 17.3 (235) | 70.1 (955) | 18.0 (245) | 11.9 (162) | 36.1 (146) | 24.8 (100) | 39.1 (158) |

^a^subscales categorized into sufficient, problematic and inadequate ability in the respective dimension: sufficient ability in this dimension 9–12, problematic ability in this dimension 7–8, inadequate ability in this dimension 3–6.

^b^n.s.: not significant
